# Land Use Eco-Efficiency and Its Convergence Characteristics Under the Constraint of Carbon Emissions in China

**DOI:** 10.3390/ijerph16173172

**Published:** 2019-08-30

**Authors:** Haoran Yang, Qun Wu

**Affiliations:** 1 College of Public Administration, Nanjing Agricultural University, Nanjing 210095, China; 2 Department of Microbiology and Plant Biology, Center for Spatial Analysis, University of Oklahoma, Norman, 73019 OK, USA

**Keywords:** land use eco-efficiency, carbon emissions, mixed directional distance function, convergence characteristics, spatial econometric model

## Abstract

By defining the connotation of land use eco-efficiency, land use eco-efficiency from 2003 to 2015 was calculated on the basis of the mixed directional distance function, and its spatial convergence analyzed using a spatial econometric model. Results showed that (1) the land use eco-efficiency in most regions of China was relatively ineffective—only Guangdong and Guangxi were relatively effective—and the spatial distribution of efficiency levels in each region was polarized. (2) Sigma and beta convergences were observed in land use eco-efficiency in China, and land use eco-efficiency in each province had an influence on the other. (3) The convergence rate of the eastern region was the same as that of the national region (0.164). The convergence rates of the central, western, and northeast regions were 0.181, 0.183, and 0.189, respectively, which were all higher than the national convergence rate. (4) Scientific and technological strength and industrial structure significantly promoted the improvement of land use eco-efficiency and steady development of land use in China.

## 1. Introduction

Land resources are valuable resources available to human beings. The rapid development of China’s economy and urban expansion while acquiring the economic wealth created by land has aggravated the excessive consumption of resources and degradation of the ecological environment and has restricted the sustainable development of society. Energy conservation and emission reduction have aroused considerable interest. People attribute the emission of carbon dioxide to the burning of fossil energy but ignore that carbon dioxide produced by land use change caused by human activities is also an important factor in global warming [1,2]. According to statistics, China became the world’s largest carbon dioxide emitter in 2007 and is expected to reach the peak of carbon emissions in 2030, thereby reducing its emission by 60–65% when compared with 2005 [3,4]. Therefore, studying the evolution rule of land use eco-efficiency in China under the constraint of carbon emissions is of great practical significance. The concept of eco-efficiency was first proposed by Schaltegger and Sturm in 1990 [5]. Eco-efficiency is the relationship between human input and output when human activities produce social wealth in the resource ecological environment on which human beings depend for survival. The rapid development of China’s economy and urban expansion have aggravated the excessive consumption of resources and the destruction of the ecological environment, and energy conservation and emission reduction have aroused widespread social concern. Studies on land use eco-efficiency have been limited, and studies on eco-efficiency mainly focus on the concept of eco-efficiency [6], evaluation system indicators [7,8], and influencing factors [9,10]. The commonly used measurement methods include the ecological footprint method [11], the factor analysis assignment method [12], the data envelopment analysis [13], super efficiency DEA [14], the three-stage DEA method [15], and nonradial distance function [16].

Convergence is derived from the mathematical concept of the tendency of two individuals to approach each other in terms of quantity or quality. Economic convergence originates from the economic convergence thought of neoclassical growth theory [17,18], which is mainly used in discussing whether economic growth or development between regions eventually tends to achieve a steady state over time. Convergence is mainly applied to the fields of finance [19] and used in determining innovation efficiency [20], resource utilization efficiency [21], and industrial land use efficiency [22] for the analysis of the dynamic evolution rule of regional differences among economic variables. Studies on the convergence analysis of eco-efficiency mainly focus on the regional differences and dynamic evolution of regional eco-efficiency [23], spatial pattern and spatial effect of energy eco-efficiency [24], and dynamic evolution of industrial eco-efficiency [25].

Basing on the research of You [26] on land use eco-efficiency, we defined land use eco-efficiency under the constraint of carbon emissions in this paper, that is driven by economic and social changes and innovations and maximizes the input and output with the minimum CO_2_ emission in the process of transforming regional land use patterns corresponding to the transition from one to another in the stage of economic and social development over a period of time. For the research content, studies on the eco-efficiency of land use are very few, the selection of indicators is mostly from the aspects of society, economy and ecology, and the policy regulation and control functions of the management of land use eco-efficiency is usually ignored. Simultaneously, very few studies have included land use carbon emissions into the efficiency of an input–output index system as an unexpected output. The estimation method that considered only the direction distance function of radial or non-radial methods overestimates or underestimates the evaluation object and the deviation between the estimation and actual results. According to Cooper et al. [27], the measurement models of efficiency are divided into radial and angular, radial and non-angular, non-radial and angular, and non-radial and non-angular. In the radial model, an input or output should vary in proportion in the evaluation of efficiency. In the angular model, a choice should be made according to the input (assuming constant output) or output (assuming constant input). In the research method of Tone [28], radial and non-radial directional distance functions are combined into mixed directional distance functions so that the defects of the original method are prevented. Hence, according to the constraint of C emissions, in this paper, we constructed the input–output system of land use eco-efficiency from the aspects of social, economic, ecological, and government management for the analysis of the evolution rule of China’s land use eco-efficiency by using a mixed directional distance function from 2003 to 2015. Meanwhile, we adopted spatial autocorrelation analysis methods to explore the spatial-temporal evolution and dynamic evolution rules of land use eco-efficiency in China and to provide a scientific basis for the promotion and steady development of land use eco-efficiency.

## 2. Methodology and Data Specification

### 2.1. Methodology

#### 2.1.1. Land Use Eco-efficiency Estimation Method

Directional distance function can solve the efficiency evaluation problem of bad output and is widely used in efficiency evaluation when an unexpected output is considered. The expression form is as follows:

(1) $$\vec{D_{0}}\left(x,y,b;g\right)=max\left\{\beta :\left(y,b\right)+\beta g\epsilon P\left(x\right)\right\},$$

where g = (y, − b) is the direction vector of output horizontal expansion, and β is the value of the directional distance function. The maximum value of the desirable output (*y*) and the minimum value of the undesirable output (*b*) are obtained by taking the set direction vector as the weight. When the radial and output angle DEA are used in the calculation of the directional distance function, the nonzero relaxation between input and output, and the efficiency measurement value becomes higher than the actual efficiency of the evaluation object. When the output angle DEA measurement efficiency cannot reflect a certain output, the input can be reduced. The non-radial and non-angular SBM (slack based model) directional distance functions are used in obtaining the non-efficiency score through the maximization of the average relaxation of all inputs and outputs. When the input or output changes in the same proportion, this method may have lower efficiency than the actual efficiency value of the evaluation object [29]. Therefore, the mixed directional distance function will be applied in this paper for the calculation of land use eco-efficiency under the constraint of land use carbon emissions in China.

Input matrix $X_{im}^{t}$ is decomposed into radial one $X_{im}^{Rt}\epsilon R_{+}^{l_{1}}$ and radial one $X_{im}^{NRt}\epsilon R_{+}^{l_{2}}\left(l_{1}+l_{2}=l\right)$, namely, $X_{im}^{t}=\left(\begin{array}{c} X_{im}^{Rt}\\ X_{im}^{NRt} \end{array}\right)$. The desirable output matrix $Y_{i}^{t}$ is decomposed into radial one $Y_{i}^{Rt}\in R_{+}^{s_{1}}$ and nonradial one $Y_{i}^{NRt}\epsilon R_{+}^{s_{2}}$ (s1 + s2 = s), namely, $Y_{i}^{t}=\left(\begin{array}{c} Y_{i}^{Rt}\\ Y_{i}^{NRt} \end{array}\right)$. Similarly, the undesirable output matrix $E_{in}^{t}$ is divided into radial one $E_{in}^{Rt}\in R_{+}^{h_{1}}$ and the non-radial part $E_{in}^{NRt}\epsilon R_{+}^{h_{2}}$ (h1 + h2 = h), namely, $E_{in}^{t}=\left(\begin{array}{c} E_{in}^{Rt}\\ E_{in}^{NRt} \end{array}\right)$. The direction vector is decomposed into six vectors, $g=\left(g_{X_{im}^{t},}g_{X_{im}^{NRt},}g_{Y_{i}^{t},}g_{Y_{i}^{Nt},\;}g_{E_{in}^{t},}g_{E_{in}^{NRt},}\right)$ and finally extended to the mixed directional distance function. The form of linear programming is as follows:

$$\vec{D_{0}}\left(X_{im}^{t},Y_{i}^{t},E_{in}^{t};g\right)=max\;w^{T}\cdot \beta$$

$$s.t\;X_{im}^{t}\lambda \leq X_{im}^{t}+\beta_{X_{im}^{Rt}}\cdot diag\left(g_{X_{im}^{Rt}}\right)$$

$$X_{im}^{NRt}\lambda \leq X_{im}^{NRt}+\beta_{X_{im}^{NRt}}\cdot diag\left(g_{X_{im}^{NRt}}\right)$$

$$Y_{i}^{Rt}\;\lambda \geq Y_{i}^{Rt}\;+\beta_{Y_{i}^{Rt}\;}\cdot diag\left(g_{Y_{i}^{Rt}}\right)$$

$$Y_{i}^{NRt}\lambda \geq Y_{i}^{NRt}+\beta_{Y_{i}^{NRt}}\cdot diag\left(g_{Y_{i}^{NRt}}\right)$$

$$E_{in}^{Rt}\lambda =E_{in}^{Rt}+\beta_{E_{in}^{Rt}}\cdot diag\left(g_{E_{in}^{Rt}}\right)$$

$$E_{in}^{NRt}\lambda =E_{in}^{NRt}+\beta_{E_{in}^{NRt}}\cdot diag\left(g_{E_{in}^{NRt}}\right)$$

$$\beta =\beta \cdot sgn{\left(\left|g\right|\right)}^{T},\;\geq 0.$$

where $w={\left(w_{X_{im}^{Rt}},w_{X_{im}^{NRt},}w_{Y_{i}^{Rt}},w_{Y_{i}^{NRt},}w_{E_{in}^{Rt}},w_{E_{in}^{NRt}}\right)}^{T}$ represents the standardized weight vector corresponding to radial input, nonradial input, radial desirable output, nonradial desirable output, radial undesirable output, and nonradial undesirable output, λ represents the weight of input X or output Y, and $\beta ={\left(\beta_{X_{im}^{Rt}},\beta_{X_{im}^{NRt},}\beta_{Y_{i}^{Rt}},\beta_{Y_{i}^{NRt},}\beta_{E_{in}^{Rt}},\beta_{E_{in}^{NRt}}\right)}^{T}\geq 0$.

#### 2.1.2. Spatial Convergence of Land Use Eco-efficiency

(1) Model of sigma convergence

Land use eco-efficiency convergence focuses on the analysis of the gap between the land use eco-efficiency levels. Land use eco-efficiency convergence indicates that the gap between the level of each region’s land use eco-efficiency gradually shrinks over time, and the regions with low land use eco-efficiency will catch up with those with high efficiency eventually. Sigma convergence is generally expressed as a standard deviation and is expressed as follows:

(2) $$S=\sqrt{\frac{\sum_{i=1}^{n}{\left(I_{i}-\frac{1}{n}\sum_{i=1}^{n}I_{i}\right)}^{2}}{n-1}}$$

where S is the standard deviation of the land use eco-efficiency, thereby indicating the extent to which the land use eco-efficiency deviates from the overall average level; $I_{i}$ represents the land use eco-efficiency value in the ith province; n is the number of provinces; and $\frac{1}{n}\overset{n}{\underset{i=1}{\sum }}I_{i}$ is China’s province average level of land use eco-efficiency.

(2) Beta convergence model

The convergence of land use eco-efficiency means that the growth rate of land use eco-efficiency of each province is negatively correlated with its level of land use eco-efficiency. Under strict assumptions, the land use eco-efficiency level of each province gradually reaches a common steady state over time, and its growth path is the same as the steady state level. The beta convergence means that the land use eco-efficiency of different regions converges to the steady state of each region. By adding other control variables, the conditional beta convergence model is obtained. The expression is as follows:

(3) $$\ln\left(\frac{I_{i,t}}{I_{i,t-1}}\right)=a+bln\left(I_{i,t-1}\right)+\lambda X+\theta_{i}+\eta_{t}+\mu_{i,t}$$

where $I_{i,t}$ represents the initial level of eco-efficiency value in the t year, $I_{i,t-1}$ represents the value of eco-efficiency in the year t−1, the time effect of $\eta_{t}$ does not vary with region, $\theta_{i}$ represents the regional effect that does not change over time, and $\mu_{i,t}$ represents the random error term. If coefficient *b* is significantly negative, then the growth rate of land use eco-efficiency is negatively correlated with its initial level. Hence, provinces with low land use eco-efficiency have a faster growth rate than those with high land use eco-efficiency. This result indicates that land use eco-efficiency in this region has a beta convergence. The convergence rate can be expressed as B=−ln(1+Tb)/T, where T is the entire time span, and the half-life of convergence can be expressed as τ=−ln(2)/ln(1+b) [30].

Ignoring spatial correlation will lead to biased estimation results. Moran’s I index can be used in determining whether the spatial correlation of land use eco-efficiency is present. If spatial correlation is present, then a spatial error or a spatial lag model can be constructed through a spatial measurement method for beta convergence. The spatial Error or Lag model is determined by the LM-lag test, the LM-error test, the Robust LM-lag test, and the Robust LM-error test [31]. Therefore, this paper proposes two hypotheses, as follows:

**Hypothesis** **1.** *Different land use eco-efficiency levels are not close and independent in geographical space distribution, that is, China’s land use eco-efficiency has significant spatial autocorrelation.*

**Hypothesis** **2.** *The regional land use eco-efficiency in China has the characteristics of beta convergence.*

### 2.2. Index Selection and Data Sources

#### 2.2.1. Index of Input and Output of Land Use Eco-efficiency

The core of land use eco-efficiency is to exchange additional economic output with low environmental impact [32]. In this study, combined with the connotation of land use eco-efficiency, input indicators from the aspects of land, capital, labor, energy and policy, and economic benefit are considered desirable output indicators, whereas ecological impact is considered the undesirable output indicator (Table 1).

(1) Input

The index of capital input is represented by fixed capital stock. Energy factor input indexes select total energy consumption. Given that this study is based on the perspective of land use carbon emissions, the selection of land input factor indexes must include the carbon sources and sinks generated in the use process of different land use types. Thus, the total land area of the province is selected as the representation. The index of labor input factors is represented by the number of employees at the end of the year. The index of policy factor input is represented by the amount of government investment in environmental pollution control.

Due to the differences in economic development levels and characteristics of various regions, this paper combined with the index system of land use eco-efficiency constructed by scholar You [31] to process the above indicators. Finally, the fixed capital stock is selected to reflect the input of capital factor, the total number of labor employment is selected to reflect the input of labor factor, the total energy consumption is selected as the input of energy factor, and the investment in environmental pollution control is selected as the input of policy factor.

(2) Desirable output

The desired output should reflect the economic value of products and services provided by the system. Therefore, the gross domestic product (GDP) of each region is taken as the desired output of land use eco-efficiency in this study.

(3) Undesirable output

Undesirable output is selected to represent CO_2_ emissions caused by land use. The types of land involved in this paper include cultivated land, construction land, woodland, garden land, grassland, water area, and unused land. The carbon sink mainly comes from the net CO_2_ from the atmosphere into the ecosystem. The carbon source comes mainly from the carbon produced by energy consumption, industrial production, transportation, and agricultural production. As the perspective of this paper is based on the carbon emissions caused by the change of land use type caused by human activities, the carbon emissions of construction land only considers the carbon emissions generated by energy consumption. Carbon source comes mainly from the carbon produced by energy consumption, industrial production, transportation and agricultural production. The carbon emissions in this paper is generated by the energy consumption of construction land as the main carbon source. We refer to the methods of the Intergovernmental Panel on Climate Change (IPCC, 2007) and the Energy Research Institute of National Development and Reform Commission in China (2003) to estimate the CO_2_ emissions generated by construction land in various provinces through relevant calculation formulas [2,33]. The calculation methods are as follows:

(4) $$E_{g}=E_{f}+E_{m}+E_{i}=G_{f}\cdot A+\left(A_{m}\cdot B+W_{m}\cdot C\right)+A_{i}\cdot D$$

(5) $$E_{k}=\sum^{\;}e_{i}=\sum^{\;}T_{i}\cdot \delta_{i}$$

(6) $$E_{t}=\sum^{\;}E_{ti}=\sum^{\;}E_{ni\cdot }\cdot \theta_{i}\cdot f_{i}$$

(7) $$E=E_{g}+E_{k}+E_{t}$$

where $E_{g}$ represents the carbon emissions of cultivated land; $E_{f}$ stands for carbon emissions from fertilizer use, $E_{m}$ represents the carbon emissions generated by the production and use of agricultural machinery, $E_{i}$ represents the carbon emissions in the irrigation process, $G_{f}\;$ is the amount of fertilizer used, $A_{m}$ represents the total sown area of crops, $W_{m}$ represents the total power of agricultural machinery; $A_{i}$ is irrigation area, $E_{k}$ represents the total carbon absorption, $e_{i}$ represents the amount of carbon absorption generated by different land use types, $T_{i\cdot }$ represents the area of the functional land type of carbon sink, $\delta_{i}$ is the carbon emission (absorption) coefficient of different land types, with positive carbon emissions and negative carbon absorption, $E_{t\;}$ represents carbon emission of construction land, $E_{ti}$ represents various energy carbon emissions, $E_{ni\cdot }$ represents the consumption of various types of energy, $\theta_{i}$ represents the coefficients of various energy transformations to standard coal, $f_{i}$ represents the carbon emissions coefficient of various energy sources, and *E* represents the net land use carbon emission.

Fossil fuels are consumed from raw coal, crude oil, and natural gas, with carbon factors of 0.7476, 0.5854, and 0.4479 tC/tce, respectively. Cultivated land is carbon source and carbon sink. The production and use of agricultural irrigation, chemical fertilizers, agricultural film, and pesticides and the transportation of agricultural machinery in the production and management process produce much more carbon emissions than carbon absorption. The carbon emission conversion coefficients (A, B, C, and D) in the process of fertilizer, seeding, usage of agricultural machinery and irrigation are 895.6 kg/t, 16.47 kg/hm^2^, 0.18 kg/kw, and 266.48 kg/hm^2^, respectively [34]. This paper only considers the carbon source of cultivated land. According to the complex land use structure characteristics in China, the factors of carbon absorption of woodland, grassland, garden, water area, and unused land are −6.44, −0.39, −6.44, −0.245, and −0.05 t/hm^2^ a, respectively [35].

The calculated standard energy carbon emission is the sum of the total consumption of various types of energy and the product of the energy conversion standard coal coefficient and the energy carbon emission coefficient. The selection of coefficients is calculated with reference to the coefficient values in the IPCC report. The carbon emissions of cultivated land are calculated by the use of chemical fertilizer, total planting area of crops, total power of agricultural machinery and irrigation area multiplied by their conversion coefficients. The carbon absorption of woodland, grassland, garden, water area, and unused land are calculated by the product of their area and corresponding coefficients.

#### 2.2.2. Index of Spatial Convergence

(1) Urbanization level. The promotion of urbanization promotes the transfer of rural labour force and population, and the increase of urban population density may have a negative impact on the ecological environment to some extent. In this study, the proportion of the employment of secondary and tertiary industries in the total employment of each region is selected to represent the urbanization rate of the region.
(2) Foreign investment. The utilization of foreign capital can achieve industrial transfer by adjusting the industrial structure of the host country and transferring high-pollution industries to the host country for production, thereby leading to the deterioration of the ecological environment of the host country, that is, the “pollution shelter” hypothesis [36]. In this study, the proportion of total foreign investment in the total regional GDP is selected as the representation.
(3) Environmental governance. As a public good provided by the government, environmental protection is noncompetitive and nonexclusive. Local governments have a good understanding of local information which is conducive for local governments to formulate effective environmental protection policies according to the behavioral preferences of local residents. Thus, the pollution control completion characterization of environmental governance situation is chosen as the representation. To prevent the occurrence of heteroscedasticity, we logically process the index data.
(4) Degree of openness. With the improvement of the degree of openness, green production can be realized in the process of absorbing advanced technologies. The proportion of total import and export in the GDP of the region is selected as the representation.
(5) Scientific and technological strength. Enterprises close to the forefront of technology are inclined to use independent innovation to achieve technological progress and efficiency growth. By contrast, enterprises far from the forefront of technology tend to improve their efficiency by catching up with technology. Therefore, the research and development (R&D) intensity of expenditure input is characterized in this study.
(6) Industrial structure. According to siphon effect, the heterogeneity of regional industrial structure will lead to the influx of factor resources from areas with high land use eco-efficiency to areas with low efficiency. The proportion of the added value of the tertiary industry is selected to represent in this study.

The data of labor force of 30 provinces and regions were obtained from the China Statistical Yearbook (2004–2016). The total energy consumption data of 30 provinces and regions except Tibet were from the China Energy Statistical Yearbook (2004–2016). Data on investment in environmental pollution control came from China’s Environmental Statistics Yearbook (2005–2016) and China’s Environmental Yearbook (2004). Land area data were obtained from the Statistical Yearbook of China’s Land and Resources (2004–2016). GDP, foreign investment, the original data and R&D expenditure intensity data were derived from the China Statistical Yearbook (2004–2016). We selected a single hero for the calculation method of fixed capital stock measurement through the GDP deflator to regional GDP deflator in 2003 for the base period of constant fixed capital stock data, and the depreciation rate was 10.96%. The capital stock of the base period is calculated by the sum of the annual growth rate and depreciation rate of the total amount of real capital formed in the five years after the base period. The foreign direct investment needed to be reduced by CPI to obtain the actual foreign direct investment based on 2003.

If the national panel data is divided into seven regions, then the panel sample quantity of each region is extremely small which will affect the accuracy of regression results. Therefore, according to the regional division method of the National Bureau of Statistics in 2011, the provinces were divided into four economic zones for analysis. The eastern region includes Shandong, Jiangsu, Hebei, Guangdong, Tianjin, Shanghai, Beijing, Fujian, Zhejiang, and Hainan; the western region includes Chongqing, Shanxi, Guizhou, Qinghai, Inner Mongolia, Ningxia and Xinjiang, Sichuan, Yunnan, and Shaanxi; the central region includes Henan, Shanxi, Hunan, Hubei, Jiangxi, and Anhui; and the northeast region includes Heilongjiang, Jilin, and Liaoning.

## 3. Results and Analysis

### 3.1. Analysis of Land Use Eco-efficiency Results

**Figure 1:**
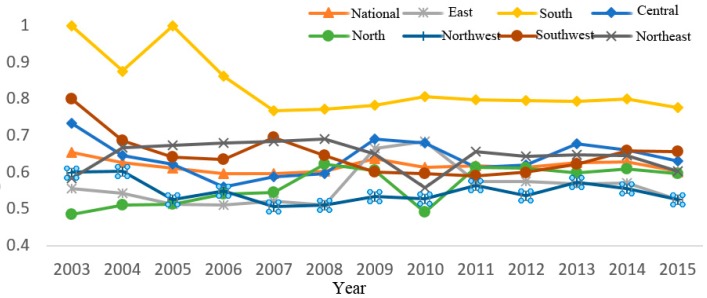
Average land use eco-efficiency in China from 2003 to 2015.

**Figure 2:**
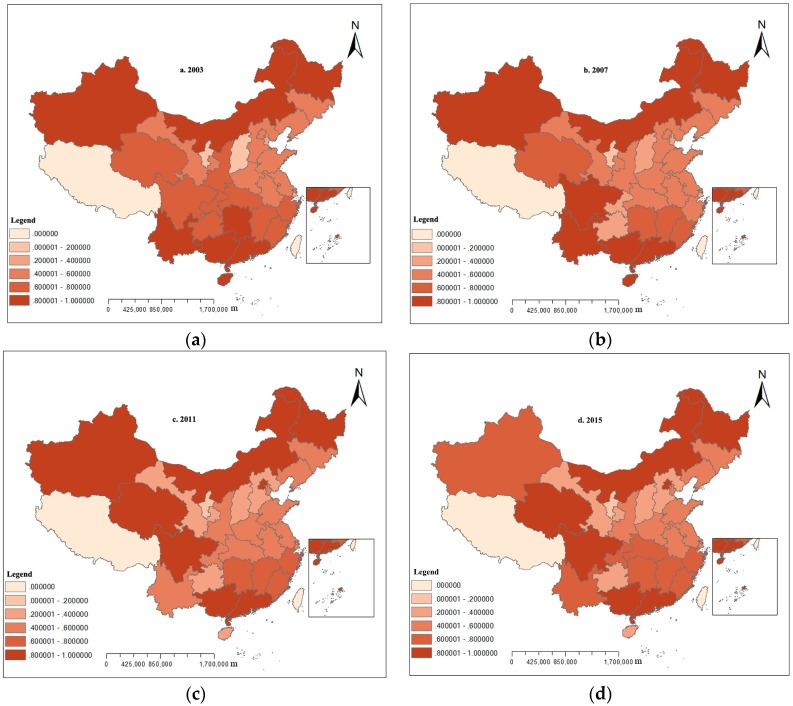
Spatial distribution of provincial land use eco-efficiency in China. (**a**–**d**) are spatial distribution maps of land use eco-efficiency in 2003, 2007, 2011, and 2015.

Using the mixed directional distance function and relevant interprovincial panel data from 2003 to 2015, the optimal production frontier of land use eco-efficiency was constructed, and the annual score of China’s inter-provincial land use eco-efficiency was calculated (see Table 2). Figure 1 shows the trend of average land use eco-efficiency changes in different regions of China from 2003 to 2015. ArcGIS software was used to draw the spatial distribution map of land use eco-efficiency in China’s provinces (see Figure 2). Figure 2a–d are spatial distribution maps of land use eco-efficiency in 2003, 2007, 2011, and 2015, respectively. China is divided into seven regions, namely, east China (Shanghai, Jiangsu, Zhejiang, Anhui, Fujian, Jiangxi and Shandong), south China (Guangdong, Hainan and Guangxi), central China (Henan, Hubei and Hunan), north China (Beijing, Tianjin, Shanxi, Hebei and Inner Mongolia), northwest China (Shaanxi, Gansu, Qinghai, Ningxia and Xinjiang), southwest China (Chongqing, Sichuan, Guizhou and Yunnan), and northeast China (Heilongjiang, Jilin and Liaoning) for discussion in this section.

As shown in Table 2, in terms of the efficiency level, the land use eco-efficiency of most regions in China from 2003 to 2015 were relatively ineffective (<1), and the efficiency values of most regions were <0.5. Only Guangdong and Guangxi were relatively effective (1). The values of land use eco-efficiency in Beijing, Jilin, Heilongjiang, Zhejiang, Fujian, Jiangxi, Hunan, Yunnan, Qinghai, and Xinjiang were >0.5.

Overall, the land use eco-efficiency in China showed a fluctuating downward trend. From 2003 to 2007, the efficiency level continued to decline. The efficiency levels continued to increase in 2008–2009 towards its peak in 2009. This result may be due to the continuous improvement of China’s economic development level (GDP growth rates of 9.6% and 10.6%), with a relatively low resource consumption and environmental pollution. The research results of this stage were essentially consistent with the research results of Yang [37] and Huang [38], thereby indicating that the fluctuation and change in the eco-efficiency of land use in China were synchronized with economic development. From 2010 to 2015, the phenomenon of repeated fluctuations decreased, thereby indicating that China’s rapid economic development and resource consumption were at the cost of ecological environment, and problems, such as deterioration of air quality and low utilization rate of resources, occurred. The emergence of these problems means the environmental pollution that is generated by human activity cannot be afforded and this will eventually lead to the decline of land use eco-efficiency.

Figure 1 shows that the average land use eco-efficiency in south China was ranked first place and it was much higher than the national average level. From 2003 to 2007, the average land use eco-efficiency in south China showed a general trend of fluctuation and decline, the average value of land use eco-efficiency from 2004 to 2006 showed the tendency of increasing at the beginning and decreasing afterward, and then the change tended to be stable after 2007. During the study period, the average efficiency of Guangdong and Guangxi in south China remained relatively effective. However, the change in land use eco-efficiency in Hainan fluctuated considerably. In 2004, the value of land use eco-efficiency reached a trough of 0.1131, which seriously affected the overall level of south China in 2004. The land use eco-efficiency in southwest China was better than the national average level, except from 2009 to 2013. The average value of land use eco-efficiency in southwest China had a considerable change. The average efficiency value continued to decrease from 2003 to 2006. After a brief recovery in 2007, the average efficiency decreased for 4 years and gradually increased from 2012 to 2015. The average value of efficiency in 2015 reached 0.6562 which exceeded the national average level. The land use eco-efficiency in central China was close to the national average level. The average efficiency of Central China gradually decreased from 2003 to 2008, increased rapidly in 2009 and 2010, and then fluctuated after 2013.

The land use eco-efficiency level in northeast China was similar to that in the whole country. From 2003 to 2008, the trend of land use eco-efficiency continued to increase, whereas the average efficiency reached a trough of 0.5587 from 2009 to 2010. However, the land use eco-efficiency level was lower than the average level in northeast China in 2003. The land use eco-efficiency in northwest and east China was lower than the national level. From 2008 to 2013, the land use eco-efficiency in the northwest region first increased and then decreased. The economy and technology in the northwest region were relatively backward and could only drive the economic development of the region by developing capital-intensive industries and becoming the pollution refuge of the whole country. Hence, the region is also an area to eliminate backward production capacity to realize ecological compensation. The improvement space of land use eco-efficiency which paid increased attention to green development and ecological protection was better than other regions. The land use eco-efficiency level in East China showed a changing trend of first falling and then rising and falling again. The initial level of land use eco-efficiency in north China was the lowest in China, and the efficiency level increased year by year until 2008. After the trough of 2010, no evident fluctuation was observed in 2015, but the final efficiency level was higher than those in north and northwest China.

Figure 2 shows the polarization of the distribution of land use eco-efficiency in China’s provinces from 2003 to 2015. The regions with high efficiency were located mainly in south, southwest, central, and northeast China, whereas the regions with low efficiency were mainly concentrated in northwest, east, and north China. The analysis results were consistent with those of Yu et al. [39] and Chu et al. [40].

### 3.2. Spatial Convergence Analysisof Land Use Eco-efficiency

#### 3.2.1. Sigma Convergence Analysis

**Figure 3:**
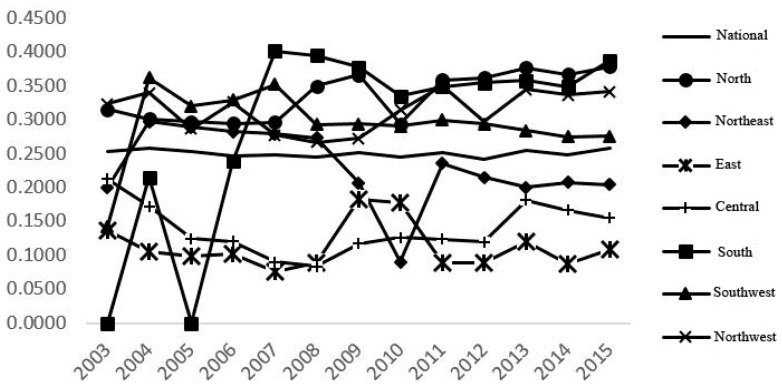
Variation of standard deviation of land use eco-efficiency value in China and seven regions.

According to Figure 3, the statistical value of the sigma convergence of China’s land use eco-efficiency fluctuated at the level of 0.25 from 2003 to 2015, thereby showing the characteristics of overall and local convergence. The standard deviation of land use eco-efficiency in north, northeast, south, southwest, and northwest China took on a trend of growth and sigma divergence during the study period, whereas the overall change in east and central China took on a tendency of declining and sigma convergence.

#### 3.2.2. Beta Convergence Analysis

We calculated the Moran’s I index of land use eco-efficiency with Geoda software to test whether a spatial autocorrelation of land use eco-efficiency exists in China. According to the geographic adjacency criterion, this paper constructed a binary spatial adjacency weight matrix. If regions *i* and *j* have a common boundary, then *W_ij_* = 1, where *W_ij_* is an element of spatial weight matrix; otherwise, *W_ij_* = 0. The results of Moran’s I index are shown in Table 3. China’s land use eco-efficiency had significant spatial negative autocorrelation, that is, different land use eco-efficiency levels were not close and independent in geographical spatial distribution, when hypothesis 1 was tenable. Therefore, the spatial econometric model was selected in the analysis of beta convergence.

**Figure 4:**
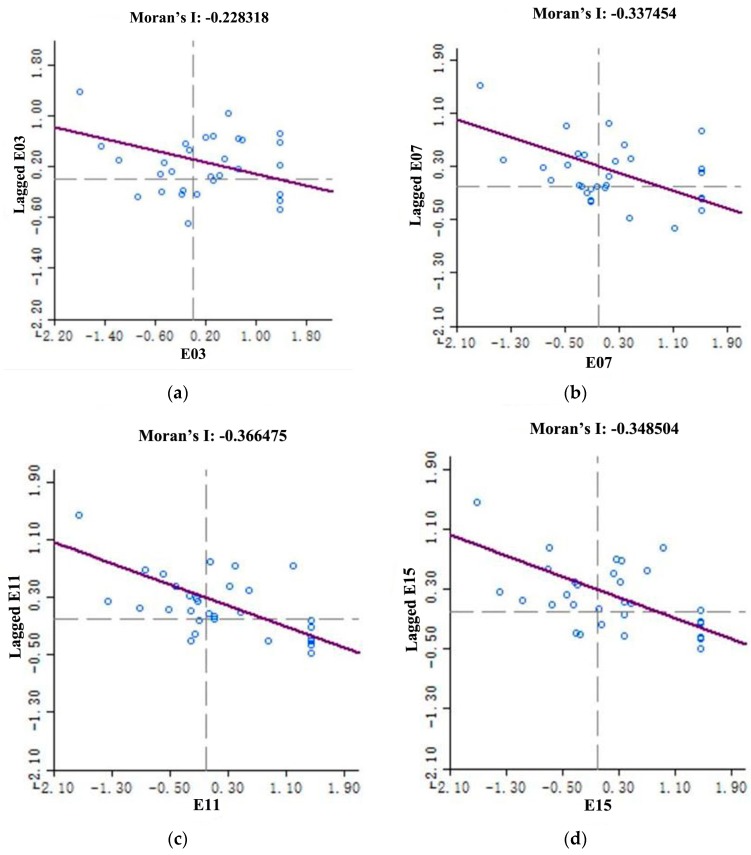
Moran scatter plots of land use eco-efficiency of provinces in China from 2003 to 2015 in selected years. (**a**–**d**) represent the Moran scatter diagrams of 2003, 2007, 2011, and 2015.

Figure 4 and Table 4 show the Moran scatter diagram and regional grouping of China’s land use eco-efficiency, respectively. Figure 4a–d represent the Moran scatter diagrams of 2003, 2007, 2011, and 2015, respectively. According to Moran scatter diagram, most regions of land use eco-efficiency values were located in the I and II quadrants, thereby showing negative spatial autocorrelation distribution characteristics. Meanwhile, only the land use eco-efficiency values of a few regions were located in the III and IV quadrants. These regions deviated from the negative spatial autocorrelation of the overall global trends which showed atypical characteristics.

Compared with the Moran scatter plots in early 2003 (Figure 4a) and late 2015 (Figure 4b), 13 provinces (43%) showed similar negative spatial autocorrelation in 2003, with 8 provinces (27%) falling in the II quadrant and 5 provinces (16%) in the IV quadrant. In 2015, 17 provinces (57%) showed similar negative spatial autocorrelation, with 10 provinces (33%) falling in the II quadrant and 7 provinces (24%) in the IV quadrant. This result indicated a significant spatial negative correlation between the land use eco-efficiency in various provinces in China. Most of the provinces showed transitional and polarization characteristics with their neighboring provinces, and a strong dependence between the provinces with high land use eco-efficiency level and the provinces with low land use eco-efficiency level was observed. The unstable and atypical regions can also be explored by Moran scatter plots, that is, provinces that deviated from the positive correlation. For instance, 17 provinces showed atypical spatial correlation, including 12 provinces in the I quadrant and 5 provinces in the III quadrant in 2003. Similarly, 12 provinces showed atypical spatial correlation, including 10 provinces falling in the I quadrant, and 2 provinces failing in the III quadrant in 2015.

Moran’s I index proved that China’s land use eco-efficiency had spatial correlation. Therefore, using the spatial effect is necessary in the analysis of the beta convergence of land use eco-efficiency and in ensuring the accuracy of regression estimation. Stata14.0 software and LM test were used to select a spatial error model and a spatial lag model. Table 5 shows that the LM test of the spatial error model was significant, whereas the spatial lag model was insignificant. Therefore, the spatial error model was selected for the beta convergence analysis of China’s land use eco-efficiency. According to the Hausman test results, the null hypothesis was significantly rejected at the 1% level. Thus, the fixed effect model was adopted.

The regression results in Table 6 shows that the regression coefficient of the core explanatory variables was significantly negative at the level of 1%, thereby indicating that β-convergence was present in land use eco-efficiency at the national, eastern, central, western and northeastern regions. Meanwhile, the result supposed the hypothesis 2 was reasonable. At the national level, the scientific and technological strength and industrial structure were significantly positive at the level of 1% and 5%, respectively. At the national level, the scientific and technological strengths and industrial structure were the key factors affecting the convergence of land use eco-efficiency. The eco-efficiency of land use between provinces tended to converge as the level of development approached. The enhancement of scientific and technological strength was conducive to the innovation of industrial advanced technology and the improvement of resource utilization efficiency, thus reducing the emission of pollutants. Therefore, strengthening scientific and technological strength can not only improve the land use eco-efficiency in one region, but also promote the efficiency of neighbouring regions. Increasing investment in scientific and technological strength is an important measure to improve the land use eco-efficiency in China in the future.

In particular, the scientific and technological strength of the eastern region was also significantly positive at the level of 5%. The reason was that the eastern region, including Beijing, Shanghai, Jiangsu, Zhejiang, Anhui and other provinces, had strong innovation ability. In contrast, the innovation pattern in other regions was relatively imperfect, the mechanism of sharing innovation resources was insufficient, and the regression coefficients were not significant, indicating that these regions also needed the provinces with strong innovation ability in the east to drive the development of the other regions, and gradually narrow the gap of efficiency levels among regions. The industrial structure coefficients of the whole country, central, western, and northeast regions were all significantly positive at the level of 10% which proved the main factors affecting the beta convergence of China’s land use eco-efficiency. However, the industrial structure and urbanization rate in the eastern region were significantly negative at the level of 5%, and the scientific and technological strengths were significantly positive, thereby indicating that the scientific and technological strengths promoted the beta convergence of the region, while the industrial structure and urbanization rate inhibit the beta convergence of the region. Urbanization is an important factor to promote the optimization of industrial structure transformation.

Compared with other regions, eastern China was economically developed, and the number of rural population migrating to the cities was much higher than other regions. The rapid growth of population brought about excessive consumption of resources and the overload of natural ecology, which inhibited the improvement of land use eco-efficiency. The coefficient of openness degree in the western region was 0.1821 and thus significantly promoted the balanced development of land use eco-efficiency in the western region at the level of 10%. The influence mechanism of the degree of openness on the land use eco-efficiency in China is complicated. According to Table 6, the regression coefficient of the openness was only significantly positive in the western region, but not in other regions. This indicated that the impact of the openness on the improvement of the land use eco-efficiency in China is uncertain, and the positive effect is not obvious. To some extent, this indicated that the western region had a good production cost advantage, which was conducive to attracting foreign capital and effectively promoting technological innovation, so as to improve the land use eco-efficiency in the western region.

## 4. Discussion

According to the concept of land use eco-efficiency under the constraint of C emissions, the values of land use eco-efficiency in China’s provinces from 2003 to 2015 were estimated by the mixed directional distance function in this paper, its sigma and beta convergence were analyzed by using the spatial econometric regression model, and the factors affecting its steady development in China were explored as well. The results showed a significant negative spatial correlation in the level of land use eco-efficiency in China, and hence hypothesis 1 was established. A spatial econometric model was constructed to prove that a beta convergence also existed, with a convergence rate of 0.164, and the growth of land use eco-efficiency among provinces had significant mutual influence, thereby demonstrating the rationality of hypothesis 2. The rates of beta convergence were 0.164, 0.181, 0.183, and 0.189. The convergence rate in eastern China was similar to that in the whole country, whereas the convergence rates in other regions were faster than that in the whole country. Scientific and technological strengths and industrial structure had a promotion in the improvement and steady development of China’s land use eco-efficiency. During the sample period, the statistical value of the land use eco-efficiency sigma convergence fluctuated at the level of 0.25, thereby showing the characteristics of overall divergence and local convergence. According to the spatial data analysis of a Moran scatter plot, the provinces were divided into four regions, namely, HH, LH, LL, and HL (HH means high land use eco-efficiency with high spatial lag, LH means low land use eco-efficiency with high spatial lag, LL means low land use eco-efficiency with low spatial lag, and HL means high land use eco-efficiency with low spatial lag) and club convergence was shown in each regional group. Land use eco-efficiency has not reached the optimal frontier in several provinces in China.

## 5. Conclusions

The results showed that industrial structure and scientific and technological strengths were important factors that improve land use eco-efficiency. Therefore, China’s industrial structure should be optimized and upgraded, and orderly industrial upgrading in the future should be promoted. First, traditional production technology should be transformed, and the promotion of energy-saving and environmental protection technology and technology should be increased for the accomplishment of clean energy production in an all-round way. Second, the tertiary industry should be developed, and related industries should be undertaken according to the local environment capacity and local conditions. Government departments should increase investment in science and technology to ensure the improvement of technological strength. At the same time, the industrialization of green production technology should be implemented as soon as possible, and the transformation of China’s industrial structure should be promoted for energy saving and green manufacturing.

## Figures and Tables

**Table 1:** Input–output indicator of land use eco-efficiency.

		Parameters	Definition of Indicator
Input indicator	Capital	Capital input per capita	Fixed capital stock/acreage
	Labor	Input of labor factors per capita	Total number of labor force/acreage
	Energy	Energy input per capita	Total energy use/acreage
	Policy	Investment in environmental protection per capita	Total amount of environmental pollution control/acreage
Output indicator	Desirable	GDP	GDP
	Undesirable	Land use carbon emissions	CO_2_

Note: GDP, gross domestic product.

**Table 2:** Land use eco-efficiency values in China from 2003 to 2015.

Province	2003	2005	2007	2009	2011	2013	2015
Beijing	0.5397	0.5468	0.6714	1	1	1	1
Tianjin	0.2854	0.2785	0.3190	0.3568	0.4163	0.4372	0.4400
Hebei	0.4097	0.4671	0.4573	0.4125	0.3871	0.3678	0.3340
Shanxi	0.1952	0.2725	0.2810	0.2506	0.2606	0.1919	0.2054
Inner Mongolia	1	1	1	1	1	1	1
Liaoning	0.4203	0.4492	0.4715	0.4839	0.4793	0.4863	0.4491
Jilin	0.5253	0.5735	0.5814	0.5823	0.5674	0.5837	0.5246
Heilongjiang	0.8076	1	1	0.8810	0.9235	0.8713	0.8350
Shanghai	0.3992	0.3942	0.4972	0.4618	0.5091	0.5062	0.4041
Jiangsu	0.5172	0.4483	0.4777	0.7031	0.5840	0.5864	0.5600
Zhejiang	0.6633	0.5839	0.5668	0.7430	0.6986	0.6967	0.6626
Anhui	0.4562	0.4803	0.4454	0.5086	0.4842	0.4166	0.4325
Fujian	0.7934	0.6588	0.6491	1	0.6713	0.7016	0.6473
Jiangxi	0.6277	0.6320	0.6103	0.7303	0.6496	0.7212	0.6099
Shandong	0.5089	0.5099	0.4973	0.5679	0.4999	0.5048	0.4457
Henan	0.5832	0.5525	0.4989	0.5531	0.4851	0.4585	0.4642
Hubei	0.7221	0.5418	0.5635	0.6928	0.5841	0.7236	0.6925
Hunan	1	0.7624	0.6768	0.7862	0.7313	0.8053	0.7594
Guangdong	1	1	1	1	1	1	1
Guangxi	1	1	1	1	1	1	1
Hainan	1	1	0.3053	0.3458	0.3958	0.3797	0.3289
Chongqing	0.6688	0.3469	0.3941	0.4828	0.5111	0.5754	0.6400
Sichuan	0.7926	1	1	1	1	1	1
Guizhou	0.7394	0.3976	0.3875	0.3093	0.2836	0.3118	0.3256
Yunnan	1	0.8220	1	0.6072	0.5626	0.6043	0.6592
Shaanxi	0.6508	0.5286	0.5293	0.5328	0.5212	0.6286	0.5486
Gansu	0.5446	0.4573	0.4402	0.3948	0.3638	0.3546	0.3160
Qinghai	0.6953	0.6667	0.5818	0.8055	1	1	1
Ningxia	0.1074	0.0998	0.1040	0.1553	0.1233	0.1187	0.1039
Xinjiang	1	0.8750	0.8748	0.7800	0.8167	0.7640	0.6624
Mean	0.6551	0.6115	0.5961	0.6376	0.6170	0.6265	0.6017

Note: Due to space constraints, only the efficiency values in odd years are shown.

**Table 3:** Land use eco-efficiency Moran’s I index in China from 2003 to 2015.

Year	Moran’s I	*p*-Value	Year	Moran’s I	*p*-Value
2003	−0.2283	0.034	2010	−0.2550	0.017
2004	−0.2919	0.050	2011	−0.3665	0.010
2005	−0.3205	0.040	2012	−0.3775	0.010
2006	−0.3355	0.020	2013	−0.3451	0.010
2007	−0.3375	0.030	2014	−0.3519	0.010
2008	−0.3640	0.020	2015	−0.3485	0.020
2009	−0.3280	0.020			

**Table 4:** Results of regional classification of land use eco-efficiency of various provinces in China from 2003 to 2015 in selected years.

Year	Type	Areas	Year	Type	Areas
2003	HH (12)	Jiangxi, Guizhou, Chongqing, Heilongjiang, Fujian, Hunan, Guangxi, Yunnan, Hubei, Sichuan, Qinghai, and Shaanxi	2011	HH (9)	Heilongjiang, Hunan, Fujian, Jilin, Jiangxi, Zhejiang, Hubei, Yunnan, and Jiangsu
	LH (8)	Gansu, Jilin, Liaoning, Hainan, Anhui, Shanghai, Shanxi, and Ningxia		LH (11)	Anhui, Chongqing, Shanghai, Liaoning, Tianjin, Gansu, Guizhou, Hainan, Ningxia, Shanxi, and Hebei
	LL (5)	Tianjin, Hebei, Jiangsu, Shandong, and Beijing		LL (3)	Shaanxi, Shandong, and Henan
	HL (5)	Zhejiang, Henan, Guangdong, Inner Mongolia, and Xinjiang		HL (7)	Xinjiang, Qinghai, Guangxi, Sichuan, Inner Mongolia, Guangdong, and Beijing
2007	HH (9)	Heilongjiang, Yunnan, Guangxi, Jilin, Fujian, Hunan, Jiangxi, Qinghai, and Zhejiang	2015	HH (10)	Heilongjiang, Hunan, Fujian, Jilin, Jiangxi, Chongqing, Yunnan, Hubei, Shaanxi, and Guangxi
	LH (8)	Liaoning, Gansu, Guizhou, Chongqing, Hainan, Shanxi, Tianjin, Ningxia, Anhui, Hebei, and Shaanxi		LH (10)	Hainan, Guizhou, Gansu, Tianjin, Liaoning, Ningxia, Shanxi, Hebei, Shanghai, and Anhui
	LL (4)	Shanghai, Jiangsu, Henan, and Shandong		LL (2)	Shandong and Henan
	HL (6)	Hubei, Beijing, Xinjiang, Guangdong, Sichuan, and Inner Mongolia		HL (7)	Sichuan, Jiangsu, Xinjiang, Beijing, Guangdong, Zhejiang, and Qinghai

**Table 5:** LM and robust LM test results of space lag effect and space error effect.

Test	Statistics	*p*-Value
LM (Lag)	1.847	0.174
Robust-LM (Lag)	1.875	0.171
LM (Error)	65.946	0.000
Robust-LM (Error)	65.974	0.000

**Table 6:** Estimation results of spatial econometric model of test for beta convergence.

Coef.	China	East	Central	West	Northeast
a	−0.5228 ** (−2.29)	−0.4736 (1.06)	−1.0784 ** (−3.42)	−1.2077 *** (−4.38)	−0.1778 (−0.38)
b	−0.5743 *** (−7.95)	−0.4349 *** (−6.74)	−0.7310 *** (−8.55)	−0.7551 *** (−6.39)	−0.8173 *** (−20.93)
X1 (ur)	−0.5340 (−1.39)	−0.8891 ** (−3.18)	0.1098 (0.13)	0.1478 (0.34)	−0.8243 ** (− 6.76)
X2 (fi)	−0.0031 (−0.75)	−0.0034 (−0.53)	−0.0148 (−1.17)	−0.0212 * (−2.18)	0.0047 (1.27)
X3 (eg)	0.0102 (1.49)	−0.0077 (−1.25)	0.0111 (−1.43)	0.0228 (1.68)	0.0005 (0.21)
X4 (op)	0.0452 (0.93)	−0.0103 (−0.22)	0.0581 (0.56)	0.1821 * (2.06)	0.1504 (1.80)
X5 (rd)	0.0675 *** (2.84)	0.0748 ** (2.70)	0.0490 (0.74)	0.0287 (0.26)	−0.0432 (−0.53)
X6 (is)	0.5148 ** (2.59)	−0.1084 ** (−0.19)	0.9706 ** (2.68)	1.1743 ** (2.80)	0.4355 * (3.45)
R^2^	0.0337	0.0071	0.0435	0.0245	0.0488
Convergence speed	0.164	0.146	0.181	0.183	0.189
F	18.03 ***	77.12 ***	73.12 ***	47.78 ***	46.47 ***

Note: (1) The standard deviation of each coefficient is shown in brackets. (2) ***, ** and * represent the significance level of 1%, 5%, and 10%, respectively.
